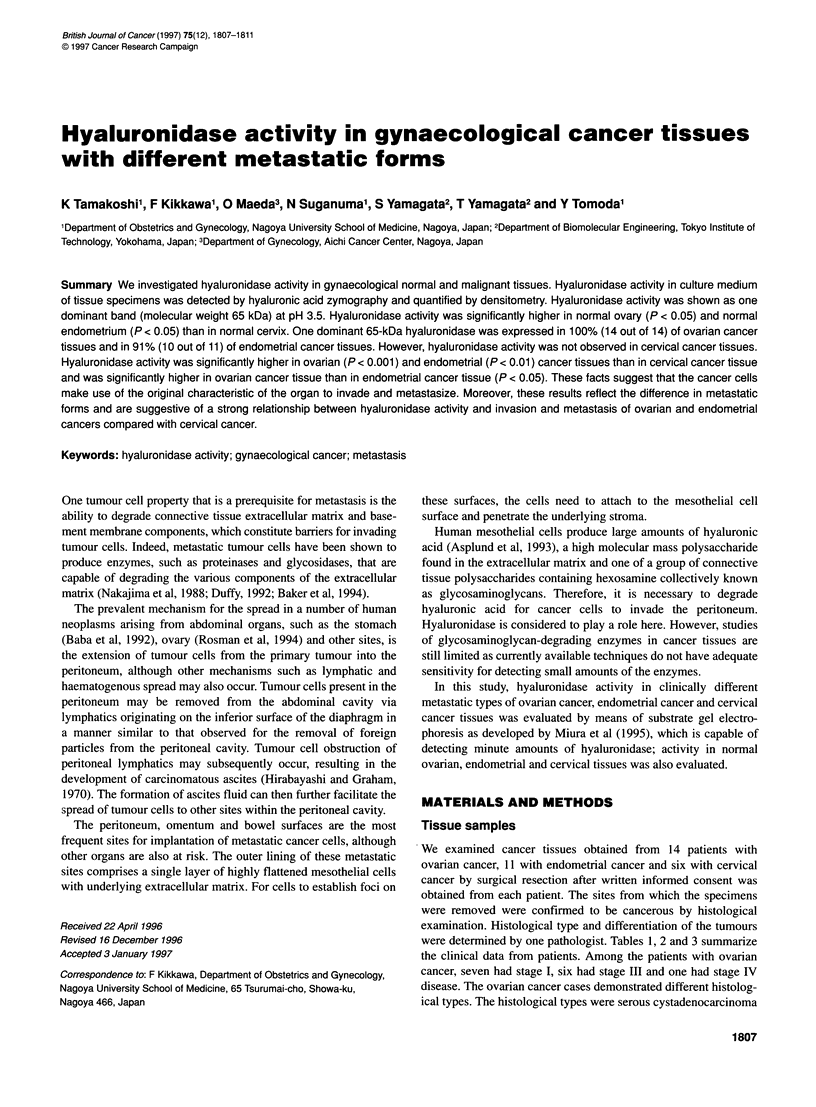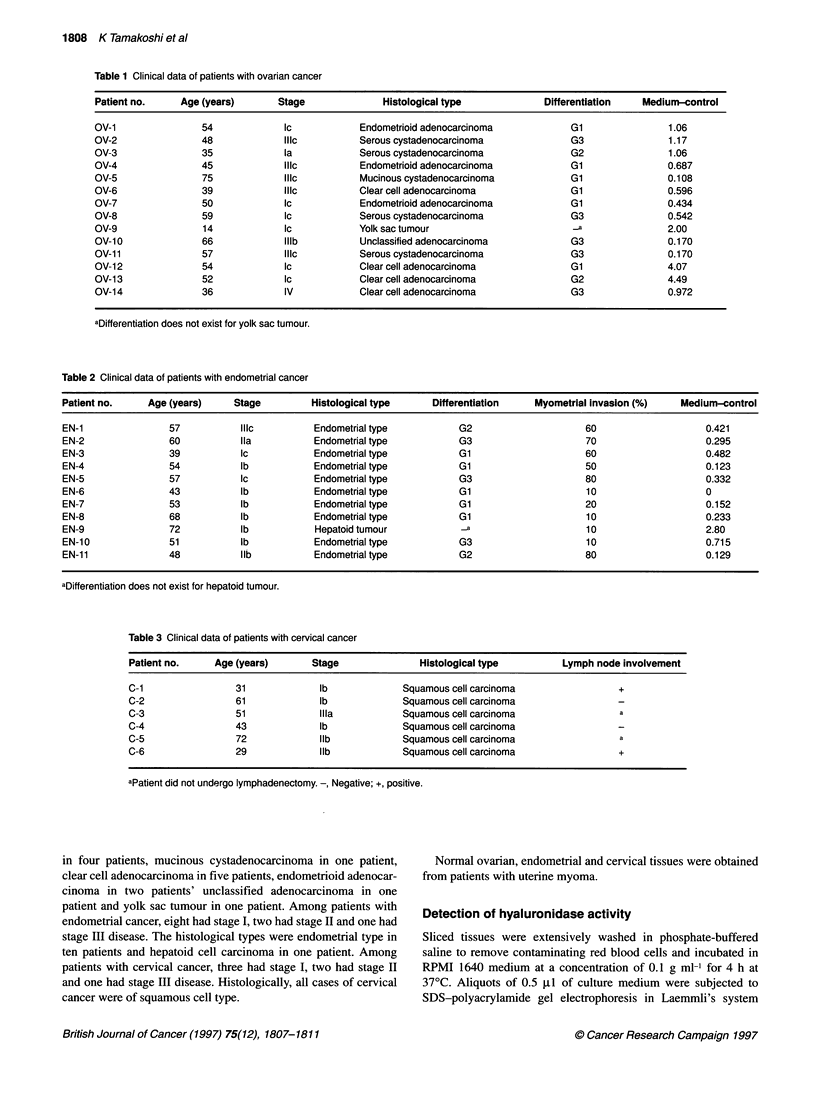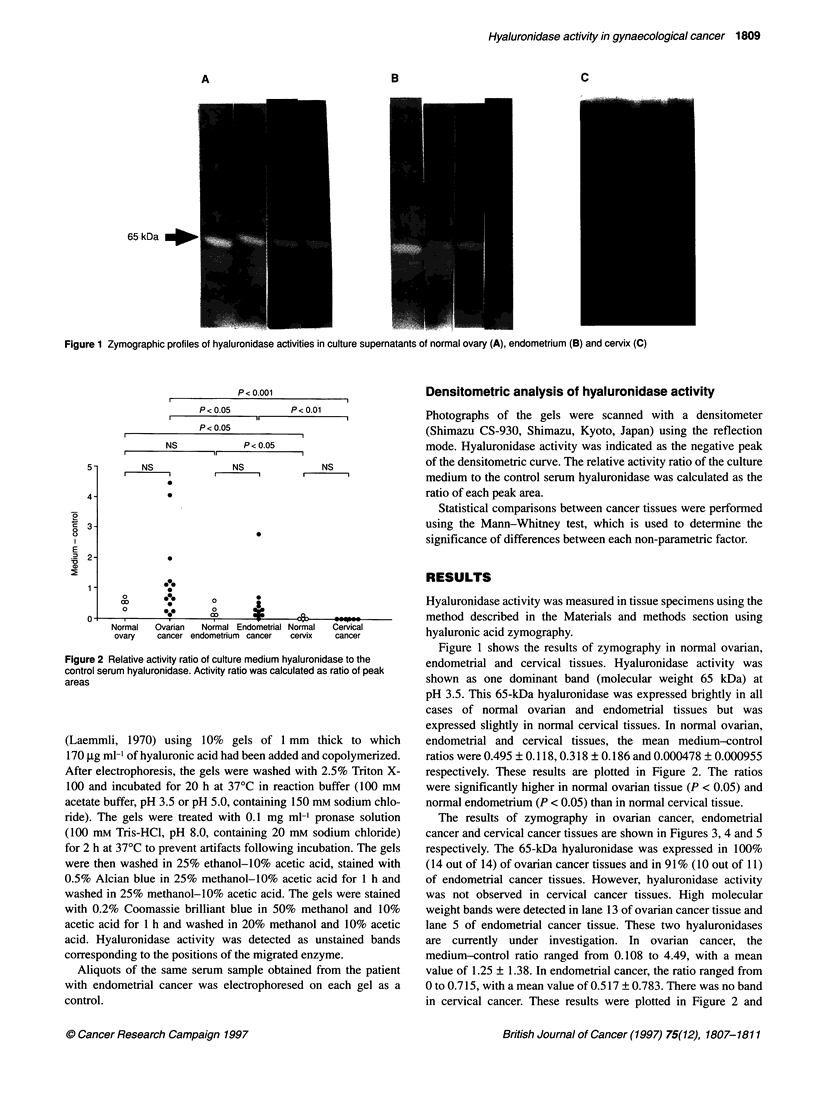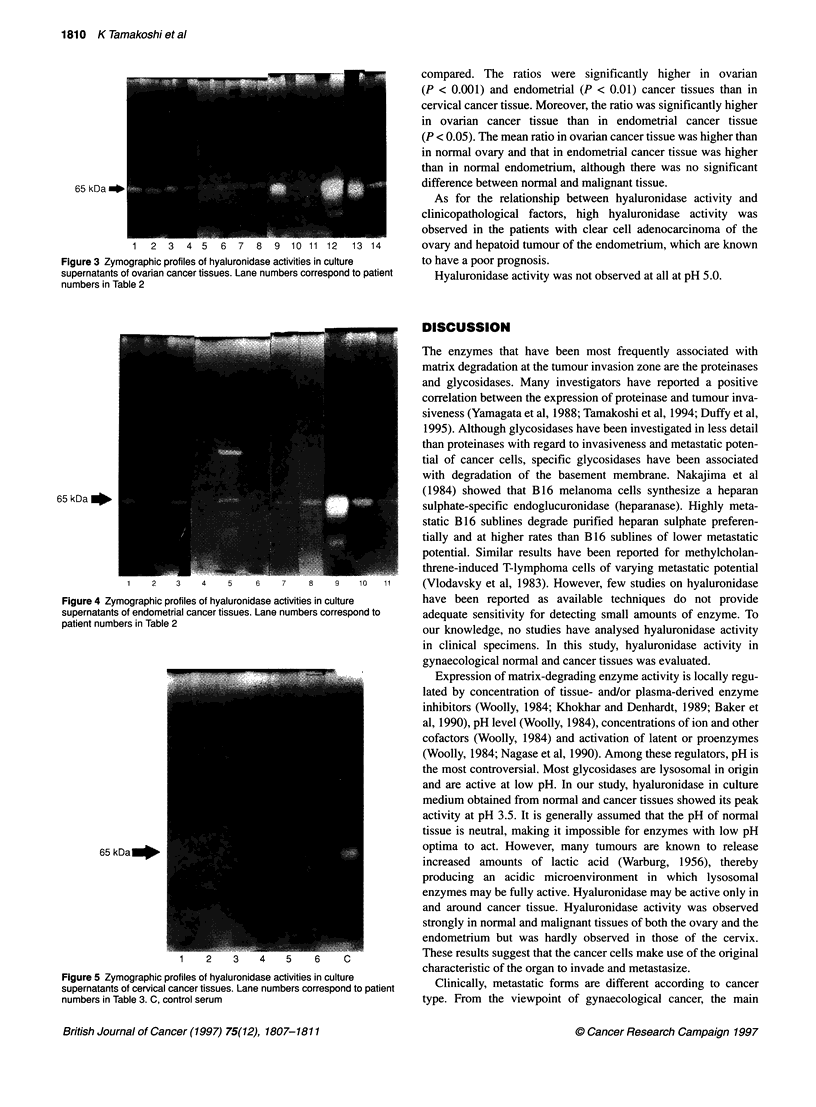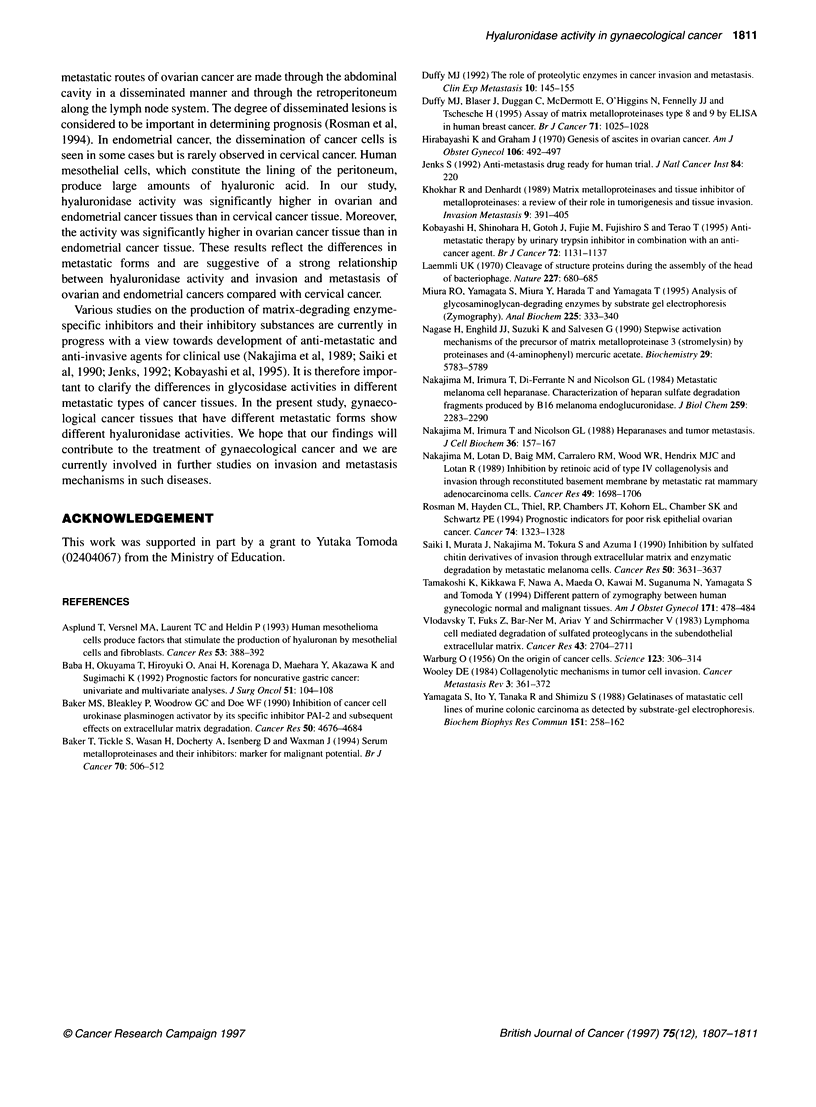# Hyaluronidase activity in gynaecological cancer tissues with different metastatic forms.

**DOI:** 10.1038/bjc.1997.308

**Published:** 1997

**Authors:** K. Tamakoshi, F. Kikkawa, O. Maeda, N. Suganuma, S. Yamagata, T. Yamagata, Y. Tomoda

**Affiliations:** Department of Obstetrics and Gynecology, Nagoya University School of Medicine, Japan.

## Abstract

**Images:**


British Journal of Cancer (1997) 75(12), 1807–1811
© 1997 Cancer Research Campaign

### Hyaluronidase activity in gynaecological cancer tissues with different metastatic forms

**K Tamakoshi[1], F Kikkawa[1], O Maeda[3], N Suganuma[1], S Yamagata[2], T Yamagata[2] and Y Tomoda[1]**

[1]Department of Obstetrics and Gynecology, Nagoya University School of Medicine, Nagoya, Japan; [2]Department of Biomolecular Engineering, Tokyo Institute of Technology, Yokohama, Japan; [3]Department of Gynecology, Aichi Cancer Center, Nagoya, Japan

**Summary** We investigated hyaluronidase activity in gynaecological normal and malignant tissues. Hyaluronidase activity in culture medium of tissue specimens was detected by hyaluronic acid zymography and quantified by densitometry. Hyaluronidase activity was shown as one dominant band (molecular weight 65 kDa) at pH 3.5. Hyaluronidase activity was significantly higher in normal ovary ($P < 0.05$) and normal endometrium ($P < 0.05$) than in normal cervix. One dominant 65-kDa hyaluronidase was expressed in 100% (14 out of 14) of ovarian cancer tissues and in 91% (10 out of 11) of endometrial cancer tissues. However, hyaluronidase activity was not observed in cervical cancer tissues. Hyaluronidase activity was significantly higher in ovarian ($P < 0.001$) and endometrial ($P < 0.01$) cancer tissues than in cervical cancer tissue and was significantly higher in ovarian cancer tissue than in endometrial cancer tissue ($P < 0.05$). These facts suggest that the cancer cells make use of the original characteristic of the organ to invade and metastasize. Moreover, these results reflect the difference in metastatic forms and are suggestive of a strong relationship between hyaluronidase activity and invasion and metastasis of ovarian and endometrial cancers compared with cervical cancer.

**Keywords:** hyaluronidase activity; gynaecological cancer; metastasis

One tumour cell property that is a prerequisite for metastasis is the ability to degrade connective tissue extracellular matrix and basement membrane components, which constitute barriers for invading tumour cells. Indeed, metastatic tumour cells have been shown to produce enzymes, such as proteinases and glycosidases, that are capable of degrading the various components of the extracellular matrix (Nakajima et al, 1988; Duffy, 1992; Baker et al, 1994).

The prevalent mechanism for the spread in a number of human neoplasms arising from abdominal organs, such as the stomach (Baba et al, 1992), ovary (Rosman et al, 1994) and other sites, is the extension of tumour cells from the primary tumour into the peritoneum, although other mechanisms such as lymphatic and haematogenous spread may also occur. Tumour cells present in the peritoneum may be removed from the abdominal cavity via lymphatics originating on the inferior surface of the diaphragm in a manner similar to that observed for the removal of foreign particles from the peritoneal cavity. Tumour cell obstruction of peritoneal lymphatics may subsequently occur, resulting in the development of carcinomatous ascites (Hirabayashi and Graham, 1970). The formation of ascites fluid can then further facilitate the spread of tumour cells to other sites within the peritoneal cavity.

The peritoneum, omentum and bowel surfaces are the most frequent sites for implantation of metastatic cancer cells, although other organs are also at risk. The outer lining of these metastatic sites comprises a single layer of highly flattened mesothelial cells with underlying extracellular matrix. For cells to establish foci on these surfaces, the cells need to attach to the mesothelial cell surface and penetrate the underlying stroma.

Human mesothelial cells produce large amounts of hyaluronic acid (Asplund et al, 1993), a high molecular mass polysaccharide found in the extracellular matrix and one of a group of connective tissue polysaccharides containing hexosamine collectively known as glycosaminoglycans. Therefore, it is necessary to degrade hyaluronic acid for cancer cells to invade the peritoneum. Hyaluronidase is considered to play a role here. However, studies of glycosaminoglycan-degrading enzymes in cancer tissues are still limited as currently available techniques do not have adequate sensitivity for detecting small amounts of the enzymes.

In this study, hyaluronidase activity in clinically different metastatic types of ovarian cancer, endometrial cancer and cervical cancer tissues was evaluated by means of substrate gel electrophoresis as developed by Miura et al (1995), which is capable of detecting minute amounts of hyaluronidase; activity in normal ovarian, endometrial and cervical tissues was also evaluated.

### MATERIALS AND METHODS

### Tissue samples

We examined cancer tissues obtained from 14 patients with ovarian cancer, 11 with endometrial cancer and six with cervical cancer by surgical resection after written informed consent was obtained from each patient. The sites from which the specimens were removed were confirmed to be cancerous by histological examination. Histological type and differentiation of the tumours were determined by one pathologist. Tables 1, 2 and 3 summarize the clinical data from patients. Among the patients with ovarian cancer, seven had stage I, six had stage III and one had stage IV disease. The ovarian cancer cases demonstrated different histological types. The histological types were serous cystadenocarcinoma

*Received 22 April 1996*
*Revised 16 December 1996*
*Accepted 3 January 1997*

*Correspondence to*: F Kikkawa, Department of Obstetrics and Gynecology, Nagoya University School of Medicine, 65 Tsurumai-cho, Showa-ku, Nagoya 466, Japan

**Table 1** Clinical data of patients with ovarian cancer

| Patient no. | Age (years) | Stage | Histological type | Differentiation | Medium–control |
|---|---|---|---|---|---|
| OV-1 | 54 | Ic | Endometrioid adenocarcinoma | G1 | 1.06 |
| OV-2 | 48 | IIIc | Serous cystadenocarcinoma | G3 | 1.17 |
| OV-3 | 35 | Ia | Serous cystadenocarcinoma | G2 | 1.06 |
| OV-4 | 45 | IIIc | Endometrioid adenocarcinoma | G1 | 0.687 |
| OV-5 | 75 | IIIc | Mucinous cystadenocarcinoma | G1 | 0.108 |
| OV-6 | 39 | IIIc | Clear cell adenocarcinoma | G1 | 0.596 |
| OV-7 | 50 | Ic | Endometrioid adenocarcinoma | G1 | 0.434 |
| OV-8 | 59 | Ic | Serous cystadenocarcinoma | G3 | 0.542 |
| OV-9 | 14 | Ic | Yolk sac tumour | –[a] | 2.00 |
| OV-10 | 66 | IIIb | Unclassified adenocarcinoma | G3 | 0.170 |
| OV-11 | 57 | IIIc | Serous cystadenocarcinoma | G3 | 0.170 |
| OV-12 | 54 | Ic | Clear cell adenocarcinoma | G1 | 4.07 |
| OV-13 | 52 | Ic | Clear cell adenocarcinoma | G2 | 4.49 |
| OV-14 | 36 | IV | Clear cell adenocarcinoma | G3 | 0.972 |

[a]Differentiation does not exist for yolk sac tumour.

**Table 2** Clinical data of patients with endometrial cancer

| Patient no. | Age (years) | Stage | Histological type | Differentiation | Myometrial invasion (%) | Medium–control |
|---|---|---|---|---|---|---|
| EN-1 | 57 | IIIc | Endometrial type | G2 | 60 | 0.421 |
| EN-2 | 60 | IIa | Endometrial type | G3 | 70 | 0.295 |
| EN-3 | 39 | Ic | Endometrial type | G1 | 60 | 0.482 |
| EN-4 | 54 | Ib | Endometrial type | G1 | 50 | 0.123 |
| EN-5 | 57 | Ic | Endometrial type | G3 | 80 | 0.332 |
| EN-6 | 43 | Ib | Endometrial type | G1 | 10 | 0 |
| EN-7 | 53 | Ib | Endometrial type | G1 | 20 | 0.152 |
| EN-8 | 68 | Ib | Endometrial type | G1 | 10 | 0.233 |
| EN-9 | 72 | Ib | Hepatoid tumour | –[a] | 10 | 2.80 |
| EN-10 | 51 | Ib | Endometrial type | G3 | 10 | 0.715 |
| EN-11 | 48 | IIb | Endometrial type | G2 | 80 | 0.129 |

[a]Differentiation does not exist for hepatoid tumour.

**Table 3** Clinical data of patients with cervical cancer

| Patient no. | Age (years) | Stage | Histological type | Lymph node involvement |
|---|---|---|---|---|
| C-1 | 31 | Ib | Squamous cell carcinoma | + |
| C-2 | 61 | Ib | Squamous cell carcinoma | – |
| C-3 | 51 | IIIa | Squamous cell carcinoma | [a] |
| C-4 | 43 | Ib | Squamous cell carcinoma | – |
| C-5 | 72 | IIb | Squamous cell carcinoma | [a] |
| C-6 | 29 | IIb | Squamous cell carcinoma | + |

[a]Patient did not undergo lymphadenectomy. –, Negative; +, positive.

in four patients, mucinous cystadenocarcinoma in one patient, clear cell adenocarcinoma in five patients, endometrioid adenocarcinoma in two patients' unclassified adenocarcinoma in one patient and yolk sac tumour in one patient. Among patients with endometrial cancer, eight had stage I, two had stage II and one had stage III disease. The histological types were endometrial type in ten patients and hepatoid cell carcinoma in one patient. Among patients with cervical cancer, three had stage I, two had stage II and one had stage III disease. Histologically, all cases of cervical cancer were of squamous cell type.

Normal ovarian, endometrial and cervical tissues were obtained from patients with uterine myoma.

### Detection of hyaluronidase activity

Sliced tissues were extensively washed in phosphate-buffered saline to remove contaminating red blood cells and incubated in RPMI 1640 medium at a concentration of 0.1 g $ml^{-1}$ for 4 h at 37°C. Aliquots of 0.5 μl of culture medium were subjected to SDS–polyacrylamide gel electrophoresis in Laemmli's system

© Cancer Research Campaign 1997

**Figure 1** Zymographic profiles of hyaluronidase activities in culture supernatants of normal ovary (**A**), endometrium (**B**) and cervix (**C**)

**Figure 2** Relative activity ratio of culture medium hyaluronidase to the control serum hyaluronidase. Activity ratio was calculated as ratio of peak areas

(Laemmli, 1970) using 10% gels of 1 mm thick to which 170 μg $ml^{-1}$ of hyaluronic acid had been added and copolymerized. After electrophoresis, the gels were washed with 2.5% Triton X-100 and incubated for 20 h at 37°C in reaction buffer (100 mM acetate buffer, pH 3.5 or pH 5.0, containing 150 mM sodium chloride). The gels were treated with 0.1 mg $ml^{-1}$ pronase solution (100 mM Tris-HCl, pH 8.0, containing 20 mM sodium chloride) for 2 h at 37°C to prevent artifacts following incubation. The gels were then washed in 25% ethanol–10% acetic acid, stained with 0.5% Alcian blue in 25% methanol–10% acetic acid for 1 h and washed in 25% methanol–10% acetic acid. The gels were stained with 0.2% Coomassie brilliant blue in 50% methanol and 10% acetic acid for 1 h and washed in 20% methanol and 10% acetic acid. Hyaluronidase activity was detected as unstained bands corresponding to the positions of the migrated enzyme.

Aliquots of the same serum sample obtained from the patient with endometrial cancer was electrophoresed on each gel as a control.

### Densitometric analysis of hyaluronidase activity

Photographs of the gels were scanned with a densitometer (Shimazu CS-930, Shimazu, Kyoto, Japan) using the reflection mode. Hyaluronidase activity was indicated as the negative peak of the densitometric curve. The relative activity ratio of the culture medium to the control serum hyaluronidase was calculated as the ratio of each peak area.

Statistical comparisons between cancer tissues were performed using the Mann–Whitney test, which is used to determine the significance of differences between each non-parametric factor.

### RESULTS

Hyaluronidase activity was measured in tissue specimens using the method described in the Materials and methods section using hyaluronic acid zymography.

Figure 1 shows the results of zymography in normal ovarian, endometrial and cervical tissues. Hyaluronidase activity was shown as one dominant band (molecular weight 65 kDa) at pH 3.5. This 65-kDa hyaluronidase was expressed brightly in all cases of normal ovarian and endometrial tissues but was expressed slightly in normal cervical tissues. In normal ovarian, endometrial and cervical tissues, the mean medium–control ratios were 0.495 ± 0.118, 0.318 ± 0.186 and 0.000478 ± 0.000955 respectively. These results are plotted in Figure 2. The ratios were significantly higher in normal ovarian tissue ($P < 0.05$) and normal endometrium ($P < 0.05$) than in normal cervical tissue.

The results of zymography in ovarian cancer, endometrial cancer and cervical cancer tissues are shown in Figures 3, 4 and 5 respectively. The 65-kDa hyaluronidase was expressed in 100% (14 out of 14) of ovarian cancer tissues and in 91% (10 out of 11) of endometrial cancer tissues. However, hyaluronidase activity was not observed in cervical cancer tissues. High molecular weight bands were detected in lane 13 of ovarian cancer tissue and lane 5 of endometrial cancer tissue. These two hyaluronidases are currently under investigation. In ovarian cancer, the medium–control ratio ranged from 0.108 to 4.49, with a mean value of 1.25 ± 1.38. In endometrial cancer, the ratio ranged from 0 to 0.715, with a mean value of 0.517 ± 0.783. There was no band in cervical cancer. These results were plotted in Figure 2 and

*© Cancer Research Campaign 1997*

**Figure 3** Zymographic profiles of hyaluronidase activities in culture supernatants of ovarian cancer tissues. Lane numbers correspond to patient numbers in Table 2

**Figure 4** Zymographic profiles of hyaluronidase activities in culture supernatants of endometrial cancer tissues. Lane numbers correspond to patient numbers in Table 2

**Figure 5** Zymographic profiles of hyaluronidase activities in culture supernatants of cervical cancer tissues. Lane numbers correspond to patient numbers in Table 3. C, control serum compared. The ratios were significantly higher in ovarian ($P < 0.001$) and endometrial ($P < 0.01$) cancer tissues than in cervical cancer tissue. Moreover, the ratio was significantly higher in ovarian cancer tissue than in endometrial cancer tissue ($P < 0.05$). The mean ratio in ovarian cancer tissue was higher than in normal ovary and that in endometrial cancer tissue was higher than in normal endometrium, although there was no significant difference between normal and malignant tissue.

As for the relationship between hyaluronidase activity and clinicopathological factors, high hyaluronidase activity was observed in the patients with clear cell adenocarcinoma of the ovary and hepatoid tumour of the endometrium, which are known to have a poor prognosis.

Hyaluronidase activity was not observed at all at pH 5.0.

### DISCUSSION

The enzymes that have been most frequently associated with matrix degradation at the tumour invasion zone are the proteinases and glycosidases. Many investigators have reported a positive correlation between the expression of proteinase and tumour invasiveness (Yamagata et al, 1988; Tamakoshi et al, 1994; Duffy et al, 1995). Although glycosidases have been investigated in less detail than proteinases with regard to invasiveness and metastatic potential of cancer cells, specific glycosidases have been associated with degradation of the basement membrane. Nakajima et al (1984) showed that B16 melanoma cells synthesize a heparan sulphate-specific endoglucuronidase (heparanase). Highly metastatic B16 sublines degrade purified heparan sulphate preferentially and at higher rates than B16 sublines of lower metastatic potential. Similar results have been reported for methylcholanthrene-induced T-lymphoma cells of varying metastatic potential (Vlodavsky et al, 1983). However, few studies on hyaluronidase have been reported as available techniques do not provide adequate sensitivity for detecting small amounts of enzyme. To our knowledge, no studies have analysed hyaluronidase activity in clinical specimens. In this study, hyaluronidase activity in gynaecological normal and cancer tissues was evaluated.

Expression of matrix-degrading enzyme activity is locally regulated by concentration of tissue- and/or plasma-derived enzyme inhibitors (Woolly, 1984; Khokhar and Denhardt, 1989; Baker et al, 1990), pH level (Woolly, 1984), concentrations of ion and other cofactors (Woolly, 1984) and activation of latent or proenzymes (Woolly, 1984; Nagase et al, 1990). Among these regulators, pH is the most controversial. Most glycosidases are lysosomal in origin and are active at low pH. In our study, hyaluronidase in culture medium obtained from normal and cancer tissues showed its peak activity at pH 3.5. It is generally assumed that the pH of normal tissue is neutral, making it impossible for enzymes with low pH optima to act. However, many tumours are known to release increased amounts of lactic acid (Warburg, 1956), thereby producing an acidic microenvironment in which lysosomal enzymes may be fully active. Hyaluronidase may be active only in and around cancer tissue. Hyaluronidase activity was observed strongly in normal and malignant tissues of both the ovary and the endometrium but was hardly observed in those of the cervix. These results suggest that the cancer cells make use of the original characteristic of the organ to invade and metastasize.

Clinically, metastatic forms are different according to cancer type. From the viewpoint of gynaecological cancer, the main

*© Cancer Research Campaign 1997*

metastatic routes of ovarian cancer are made through the abdominal cavity in a disseminated manner and through the retroperitoneum along the lymph node system. The degree of disseminated lesions is considered to be important in determining prognosis (Rosman et al, 1994). In endometrial cancer, the dissemination of cancer cells is seen in some cases but is rarely observed in cervical cancer. Human mesothelial cells, which constitute the lining of the peritoneum, produce large amounts of hyaluronic acid. In our study, hyaluronidase activity was significantly higher in ovarian and endometrial cancer tissues than in cervical cancer tissue. Moreover, the activity was significantly higher in ovarian cancer tissue than in endometrial cancer tissue. These results reflect the differences in metastatic forms and are suggestive of a strong relationship between hyaluronidase activity and invasion and metastasis of ovarian and endometrial cancers compared with cervical cancer.

Various studies on the production of matrix-degrading enzyme-specific inhibitors and their inhibitory substances are currently in progress with a view towards development of anti-metastatic and anti-invasive agents for clinical use (Nakajima et al, 1989; Saiki et al, 1990; Jenks, 1992; Kobayashi et al, 1995). It is therefore important to clarify the differences in glycosidase activities in different metastatic types of cancer tissues. In the present study, gynaecological cancer tissues that have different metastatic forms show different hyaluronidase activities. We hope that our findings will contribute to the treatment of gynaecological cancer and we are currently involved in further studies on invasion and metastasis mechanisms in such diseases.

### ACKNOWLEDGEMENT

This work was supported in part by a grant to Yutaka Tomoda (02404067) from the Ministry of Education.

### REFERENCES

Asplund T, Versnel MA, Laurent TC and Heldin P (1993) Human mesothelioma cells produce factors that stimulate the production of hyaluronan by mesothelial cells and fibroblasts. *Cancer Res* **53**: 388–392

Baba H, Okuyama T, Hiroyuki O, Anai H, Korenaga D, Maehara Y, Akazawa K and Sugimachi K (1992) Prognostic factors for noncurative gastric cancer: univariate and multivariate analyses. *J Surg Oncol* **51**: 104–108

Baker MS, Bleakley P, Woodrow GC and Doe WF (1990) Inhibition of cancer cell urokinase plasminogen activator by its specific inhibitor PAI-2 and subsequent effects on extracellular matrix degradation. *Cancer Res* **50**: 4676–4684

Baker T, Tickle S, Wasan H, Docherty A, Isenberg D and Waxman J (1994) Serum metalloproteinases and their inhibitors: marker for malignant potential. *Br J Cancer* **70**: 506–512

Duffy MJ (1992) The role of proteolytic enzymes in cancer invasion and metastasis. *Clin Exp Metastasis* **10**: 145–155

Duffy MJ, Blaser J, Duggan C, McDermott E, O'Higgins N, Fennelly JJ and Tschesche H (1995) Assay of matrix metalloproteinases type 8 and 9 by ELISA in human breast cancer. *Br J Cancer* **71**: 1025–1028

Hirabayashi K and Graham J (1970) Genesis of ascites in ovarian cancer. *Am J Obstet Gynecol* **106**: 492–497

Jenks S (1992) Anti-metastasis drug ready for human trial. *J Natl Cancer Inst* **84**: 220

Khokhar R and Denhardt (1989) Matrix metalloproteinases and tissue inhibitor of metalloproteinases: a review of their role in tumorigenesis and tissue invasion. *Invasion Metastasis* **9**: 391–405

Kobayashi H, Shinohara H, Gotoh J, Fujie M, Fujishiro S and Terao T (1995) Anti-metastatic therapy by urinary trypsin inhibitor in combination with an anti-cancer agent. *Br J Cancer* **72**: 1131–1137

Laemmli UK (1970) Cleavage of structure proteins during the assembly of the head of bacteriophage. *Nature* **227**: 680–685

Miura RO, Yamagata S, Miura Y, Harada T and Yamagata T (1995) Analysis of glycosaminoglycan-degrading enzymes by substrate gel electrophoresis (Zymography). *Anal Biochem* **225**: 333–340

Nagase H, Enghild JJ, Suzuki K and Salvesen G (1990) Stepwise activation mechanisms of the precursor of matrix metalloproteinase 3 (stromelysin) by proteinases and (4-aminophenyl) mercuric acetate. *Biochemistry* **29**: 5783–5789

Nakajima M, Irimura T, Di-Ferrante N and Nicolson GL (1984) Metastatic melanoma cell heparanase. Characterization of heparan sulfate degradation fragments produced by B16 melanoma endoglucuronidase. *J Biol Chem* **259**: 2283–2290

Nakajima M, Irimura T and Nicolson GL (1988) Heparanases and tumor metastasis. *J Cell Biochem* **36**: 157–167

Nakajima M, Lotan D, Baig MM, Carralero RM, Wood WR, Hendrix MJC and Lotan R (1989) Inhibition by retinoic acid of type IV collagenolysis and invasion through reconstituted basement membrane by metastatic rat mammary adenocarcinoma cells. *Cancer Res* **49**: 1698–1706

Rosman M, Hayden CL, Thiel, RP, Chambers JT, Kohorn EL, Chamber SK and Schwartz PE (1994) Prognostic indicators for poor risk epithelial ovarian cancer. *Cancer* **74**: 1323–1328

Saiki I, Murata J, Nakajima M, Tokura S and Azuma I (1990) Inhibition by sulfated chitin derivatives of invasion through extracellular matrix and enzymatic degradation by metastatic melanoma cells. *Cancer Res* **50**: 3631–3637

Tamakoshi K, Kikkawa F, Nawa A, Maeda O, Kawai M, Suganuma N, Yamagata S and Tomoda Y (1994) Different pattern of zymography between human gynecologic normal and malignant tissues. *Am J Obstet Gynecol* **171**: 478–484

Vlodavsky T, Fuks Z, Bar-Ner M, Ariav Y and Schirrmacher V (1983) Lymphoma cell mediated degradation of sulfated proteoglycans in the subendothelial extracellular matrix. *Cancer Res* **43**: 2704–2711

Warburg O (1956) On the origin of cancer cells. *Science* **123**: 306–314

Wooley DE (1984) Collagenolytic mechanisms in tumor cell invasion. *Cancer Metastasis Rev* **3**: 361–372

Yamagata S, Ito Y, Tanaka R and Shimizu S (1988) Gelatinases of matastatic cell lines of murine colonic carcinoma as detected by substrate-gel electrophoresis. *Biochem Biophys Res Commun* **151**: 258–162

© Cancer Research Campaign 1997